# Supplementary material for: Identification of photoperiod-induced specific miRNAs in the adrenal glands of Sunite sheep (Ovis aries)
Source: Front Vet Sci. 2022 Jul 22;9:888207. doi: 10.3389/fvets.2022.888207 (PMC9354845; doi:10.3389/fvets.2022.888207)
Supplement: Supplementary file 1 [file Data_Sheet_1.ZIP › Supplementary file/miRDeep2 file.zip!/miRDeep2 file/Know_Structure/LP42A1/oar-mir-218a.pdf]

miRBase precursor : oar-mir-218a
Total read count : 46830
oar-miR-218a read count : 46829
remaining reads : 1

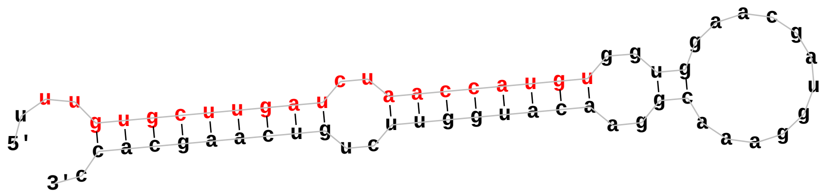

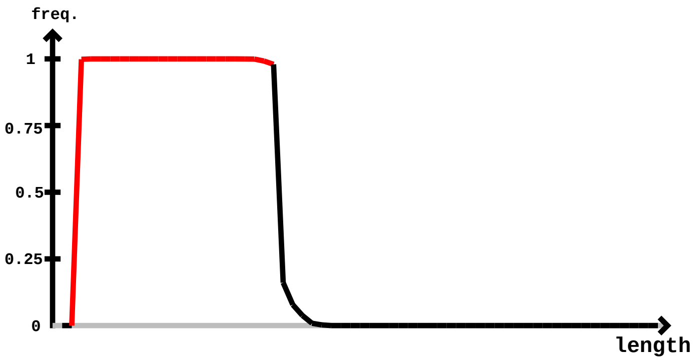

oar-miR-218a

```
5'- uuugugcuugaucuaaccaugugguggaacgauggaaacggaacaugguucugucaagcacc   -3'    exp
    ...(((((((((..((((((((..((............))..))))))))..))))))))).    reads      mm         sample
    uuugugcuugaucuaaccaugu........................................    2          0          seq
    .uugugcuugaucuaacc............................................    11         0          seq
    .uugugcuugaucuaacca...........................................    19         0          seq
    .uugugcuugaucuaaccau..........................................    344        0          seq
    .uugugcuugaucuaaccaug.........................................    580        0          seq
    .uugugcuugaucuaaccaugu........................................    38229      0          seq
    .uugugcuugaucuaacAaugu........................................    100        1          seq
    .uugugcuugaucuaaccaugug.......................................    3868       0          seq
    .uugugcuugaucuaaccaugugg......................................    1852       0          seq
    .uugugcuugaucuaaccauguggu.....................................    1361       0          seq
    .uugugcuugaucuaaccauguggU....................................    268        1          seq
    .uugugcuugaucuaaccauguggug....................................    7          0          seq
    .uugugcuugaucuaaccauguggUg...................................    118        1          seq
    .uugugcuugaucuaaccaugugggg...................................    1          0          seq
    ..ugugcuugaucuaaccaugu........................................    9          0          seq
    ..ugugcuugaucuaaccaugug.......................................    1          0          seq
    ..ugugcuugaucuaaccaugugg......................................    40         0          seq
    ..ugugcuugaucuaaccauguggu.....................................    8          0          seq
    ..ugugcuugaucuaaccauguggU....................................    4          1          seq
    ..ugugcuugaucuaaccauguggUg...................................    1          1          seq
    ...gugcuugaucuaaccaugu........................................    2          0          seq
    ...gugcuugaucuaaccaugug.......................................    1          0          seq
    ....ugcuugaucuaaccauguggU....................................    1          1          seq
    .....gcuugaucuaaccaugu........................................    1          0          seq
    .....gcuugaucuaaccaugugg......................................    1          0          seq
    ........................uggaacgauggaaacggaacaugg..............    1          0          seq
```
